# Clinical Setting Does Not Impact Baseline Patient Reported Outcomes Measures in Patients Undergoing Anterior Cervical Diskectomy and Fusion: A Prospective Study

**DOI:** 10.3390/jcm14165852

**Published:** 2025-08-19

**Authors:** Rohan Gopinath, Rohan I. Suresh, Hershil Patel, Ivan B. Ye, Alexandra E. Thomson, Jacob Bruckner, Julio J. Jauregui, Ali A. Aneizi, Louis J. Bivona, Daniel L. Cavanaugh, Eugene Y. Koh, R. Frank Henn, Daniel Gelb, Steven C. Ludwig

**Affiliations:** Division of Spine Surgery, Department of Orthopaedics, University of Maryland School of Medicine, Baltimore, MD 21201, USA

**Keywords:** anterior cervical discectomy and fusion, patient-reported outcome measures, PROMIS, neck disability index, preoperative assessment, timing of assessment

## Abstract

**Background/Objectives**: Patient-reported outcome measures (PROMs) are widely used tools in orthopedic surgery for evaluating clinical outcomes, guiding research, and supporting value-based care. However, the optimal timing for collecting baseline PROMs, whether in clinic prior to surgery or on the day of surgery, remains uncertain. This study investigated whether the clinical setting (preoperative clinic vs. day of surgery) affects baseline PROMs in patients undergoing anterior cervical discectomy and fusion (ACDF). **Methods**: Patients undergoing elective, primary ACDF at a single institution between August 2019 and June 2021 were prospectively enrolled. Inclusion criteria included age over 18, English literacy, and eligibility for primary ACDF. Participants completed PROMIS domains, Neck Disability Index (NDI), Modified Japanese Orthopaedic Association (mJOA) score, and Visual Analog Scale (VAS) at two time points: during the preoperative clinic visit and again in the perioperative area on the day of surgery. A subgroup analysis was performed for patients with anxiety, defined as a PROMIS anxiety score ≥ 59.4. **Results**: A total of 63 patients were enrolled, with 48 completing both sets of surveys. The average time between the two assessments was 7.9 days (95% CI: 6.4–9.3). After Bonferroni correction (α = 0.005), no significant differences were observed in any PROMs across the two time points, including PROMIS physical function (*p* = 0.398), pain interference (*p* = 0.682), fatigue (*p* = 0.019), social satisfaction (*p* = 0.331), anxiety (*p* = 0.047), depression (*p* = 0.042), NDI (*p* = 0.072), mJOA (*p* = 0.566), VAS neck pain (*p* = 0.054), or VAS overall pain (*p* = 0.335). Subgroup analysis of anxious patients similarly revealed no statistically significant changes between settings. **Conclusions**: PROM scores were not meaningfully different between the preoperative clinic and the day of surgery in patients undergoing ACDF. These results are most applicable when baseline PROMs are collected within about one week of ACDF. These findings support flexibility in workflow design for PROM collection, as the timing and setting do not significantly impact baseline responses.

## 1. Introduction

Patient-reported outcome measures (PROMs) have become integral to modern surgical care because they capture the patient’s subjective experience of pain, function, and quality of life in a quantifiable manner. PROMs are tools that have been validated for use in orthopedic surgery to assess the effectiveness of surgical interventions [1,2,3,4,5]. These instruments have been widely adopted in national registries, quality improvement initiatives, and bundled payment models to monitor treatment effectiveness and guide clinical decision-making [3]. With the growing emphasis on providing value-based care, PROMs have been implemented into clinical care, research, and cost-effectiveness analysis, particularly in spine surgery [6]. PROMs are often the primary outcome measure in spine research, replacing conventional outcomes such as radiographic findings and reoperation rates [7,8]. In addition to the research utility, baseline PROMs are critical for assessing postoperative improvement, determining minimal clinically important differences, and informing preoperative counseling. In particular, the effectiveness of anterior cervical discectomy and fusion (ACDF) in the treatment of degenerative pathology has been frequently studied using PROMs in the literature [9,10,11,12,13].

Despite their widespread use, logistical challenges remain regarding when to administer baseline surveys. The optimal timing for obtaining baseline PROMs prior to surgery remains unclear. PROMs can be collected at the preoperative clinic visit, a time point when patients have decided to pursue surgical treatment [14]. Technology-enabled engagement platforms integrate PROMs into routine care and can support patient-timed completion outside the clinic [15]. These surveys can be implemented as part of the surgical planning workflow. Furthermore, patients can complete the surveys while waiting in clinic. However, given the variation in time between the preoperative clinic visit and day of surgery, it is unknown if this measurement is the most accurate representation of their baseline status. Another strategy is to collect baseline PROMs on the same day as the surgery which would most accurately resemble the patient’s current state of health. However, collecting PROMs on the day of surgery encounters various potential challenges. Filling out PROMs is a time intensive process and thus requires significant coordination with the surgical staff who have various clinical duties during the perioperative period. In a busy preoperative surgical unit, PROM collection using this method might not be feasible. Furthermore, anxiety and stress about the upcoming surgery, particularly in cervical spine surgery, may impact the recorded responses, raising questions about the accuracy of the PROMs collected on the day of surgery. Finally, asking patients to recall their preoperative health status after surgery has been investigated as an option. While these results are potentially subject to memory and recall bias, studies have demonstrated preoperative PROMs have reasonable accuracy up to six weeks postoperatively [16,17]. Variations in institutional workflow, staffing, and patient availability mean that some centers collect PROMs exclusively during preoperative consultations, while others obtain them on the day of surgery to reflect the patient’s most current status. However, the day-of-surgery environment may influence responses due to situational anxiety, time pressure, or acute symptom fluctuations. Conversely, surveys collected in advance may not capture symptom progression that occurs between consultation and surgery.

It remains unclear if there are any differences in collecting baseline PROMs preoperatively in the clinic setting or on the same day of the surgery. The primary purpose of this study is to compare the PROMs collected both at the preoperative clinic visit and perioperatively on the same day of ACDF surgery. The secondary objective is to assess if anxious patients have differences in baseline PROMs between the two collection time points. Using this prospective study on patients undergoing elective ACDF, we hypothesize that PROMs obtained on the day of surgery will be different than those obtained earlier at the preoperative clinic visit. Determining whether timing affects results is crucial for preserving data integrity, especially in institutions that rely on PROMs as primary outcome measures for research or assessing clinical benchmarks.

## 2. Methods

### 2.1. Patient Selection and Data Collection

Institutional Review Board approval (HP-00062261) was obtained to prospectively enroll and consent consecutive patients undergoing ACDF at an urban tertiary care academic center between August 2019 and June 2021. Adults (18 years or older) undergoing elective, primary one- or two-level ACDF were eligible. Eligibility required English-language literacy because the study instruments (PROMIS, NDI, mJOA, VAS) and routine clinical workflow at our site were implemented in English during the study period. Patients were excluded if they were undergoing revision ACDF or combined anterior and posterior cervical surgery. We did not prospectively track the number of patients excluded for language during screening. Although validated translations exist for several instruments, during the study period our Research Electronic Data Capture (REDCap) platform (Vanderbilt University, Nashville, TN, USA) and clinic workflows supported English-only administration. Interpreter-supported or translated administration was not implemented due to operational and licensing constraints. Patients completed the Patient-Reported Outcomes Measurement Information System (PROMIS), Neck Disability Index (NDI), Modified Japanese Orthopaedic Association (mJOA), and the Visual Analog Scale (VAS on two occasions: once at the preoperative clinic appointment, approximately 1 week prior to surgery and once in the perioperative holding area on the same day of surgery.

PROMs collection was integrated into both clinical and perioperative workflows via the REDCap platform, accessible on secure, password-protected tablets. In the preoperative clinic, surveys were administered in a dedicated examination room before evaluation by the spine surgeon. On the day of surgery, patients completed surveys in the preoperative holding area, typically within 60–90 min prior to anesthesia induction. A research coordinator or trained clinical staff member was present to provide instructions and assist with technical issues; however, no coaching regarding responses was given.

The PROMIS domains were administered as computerized adaptive tests (CATs), which select subsequent questions based on prior responses, reducing survey burden while maintaining measurement precision. The NDI, mJOA, and VAS were presented in fixed question formats. Patients were encouraged to answer all questions; incomplete surveys were flagged in real time, allowing the administrator to prompt completion.

Demographic information, such as age, sex, race, smoking status, and surgical history, were inputted by the patient into the REDCap form. Body mass index (BMI) and the Charlson comorbidity index (CCI) were obtained from chart review of the patients’ electronic medical record (EMR). Total number of surgeries was defined as the cumulative count of prior operative procedures of any type before the index ACDF, excluding the index ACDF.

### 2.2. Outcomes

The primary outcome measures included the 6 domains of PROMIS: Physical Function, Pain Interference, Fatigue, Social Satisfaction, Anxiety, and Depression as well as the NDI, mJOA, and VAS for neck and body pain.

### 2.3. Statistical Analysis

Data was collected using REDCap and managed using Microsoft Excel (Microsoft, Redmond, WA, USA). Mean, standard deviation, median, and 95% confidence intervals (CI) were calculated for continuous variables. Frequencies were computed for categorical variables. The primary analysis was a comparison of the PROMs between the two time points using a two-tailed paired *t*-test. The secondary analysis on the subgroup of patients with anxiety assessed if having anxiety was associated with a difference in PROMs between the two settings. Anxiety was defined as having a PROMIS anxiety score ≥ 59.4 at either time point, which was correlated to a General Anxiety Disorder-7 (GAD-7) score of greater than 8 [18,19]. Chi-square analysis and Fisher’s exact test were used to evaluate relationships between categorical variables. Student’s *T*-test was used to evaluate continuous variables with normal distribution and Wilcoxon’s rank-sum test was used as the non-parametric alternative for continuous variables.

An a priori alpha was set at 0.05 for statistical significance. To control familywise type I error for the primary cross-setting comparisons, we applied a Bonferroni correction with α = 0.05/k, treating the ten pre-specified domain tests (six PROMIS domains plus NDI, mJOA, VAS-neck, VAS-overall) as one family; thus α = 0.005, two-sided. An a priori power analysis using the minimal clinically important difference (MCID) for the various outcome measures determined that at least 48 patients are needed to achieve power of 0.8 for this study [20,21]. With an estimated dropout rate of 15%, 57 patients would need to be enrolled. All statistical analyses were performed using SAS v9.4 statistical software (SAS Institute, Cary, NC, USA).

## 3. Results

In total, 63 patients were enrolled in this study, of which 48 patients completed both surveys and were included in the final analysis. The study population had a mean age of 52.4 years, BMI of 31.4 kg/m^2^, and CCI of 1.3 points, and was majority female (58.3%), white (75.0%), and never-smokers (54.2%; Table 1). The mean amount of time between the preoperative clinic visit and the actual surgery was 7.9 days (95% CI 6.4–9.3 days). Further demographic characteristics of the overall study cohort are presented in Table 1.

The difference in between preoperative clinic PROMs and same day of surgery PROMs for PROMIS Physical Function (39.2 versus 39.8, *p* = 0.398), Pain Interference (64.5 versus 64.2, *p* = 0.682), Fatigue (60.5 versus 58.5, *p* = 0.019), Social Satisfaction (40.1 versus 39.2, *p* = 0.331), Anxiety (60.0 versus 58.6, *p* = 0.047), and Depression (53.7 versus 51.7, *p* = 0.042) did not reach the Bonferroni-corrected threshold for statistical significance (Table 2). Similarly, there was no statistical difference in NDI (22.3 versus 20.8, *p* = 0.072), mJOA (14.8 versus 15.0, *p* = 0.566), VAS neck pain (6.0 versus 5.4, *p* = 0.054), and VAS overall pain (5.2 versus 4.8, *p* = 0.335).

In the secondary analysis, patients with anxiety were more often female than patients without anxiety (68.8% versus 37.5%, *p* = 0.038; Table 3). However, there was no difference in age (*p* = 0.255), race (0.067), smoking status (*p* = 0.341), BMI (*p* = 0.768), and CCI (*p* = 0.063). Of the anxiety patients, there was similarly no statistical difference in any PROMs between clinic and day of surgery: PROMIS Physical Function (36.5 versus 37.3, *p* = 0.348), Pain Interference (66.6 versus 66.6, *p* = 0.949), Fatigue (63.8 versus 62.3, *p* = 0.097), Social Satisfaction (37.9 versus 37.3, *p* = 0.628), Anxiety (65.1 versus 63.2, *p* = 0.044), and Depression (57.2 versus 55.2, *p* = 0.034), NDI (26.0 versus 24.1, *p* = 0.079), mJOA (14.2 versus 14.6, *p* = 0.328), VAS neck pain (6.6 versus 6.2, *p* = 0.263), and VAS overall pain (6.2 versus 5.7, *p* = 0.301; Table 4).

## 4. Discussion

Obtaining accurate baseline PROMs is important to evaluate changes in PROMs following spine surgeries. However, collecting PROMs on the day of surgery is challenging due to logistics and the anxiety of surgery, making the preoperative clinic visit a more feasible option. In this prospective study of 48 patients, there were no statistical differences in any PROMs between the preoperative clinic and day of surgery. Furthermore, the observed negligible differences in PROMs did not approach the MCID for any of the PROMs [20,21] and are not clinically relevant. Therefore, this study provides evidence that baseline PROMs can be obtained at either setting.

Our study is the first to evaluate the validity of baseline PROMs in spine surgery, presenting prospective data on 48 patients all undergoing ACDF surgery with a short time interval between the preoperative clinic visit and day of surgery with a mean of 8 days. The paucity of prior studies on baseline PROMs has been reported in sports literature. Bryant et al. studied 177 patients undergoing anterior cruciate ligament reconstruction and compared PROMs completed 4 weeks prior to surgery to those collected on the day of surgery [22]. They found a moderate to strong correlation between the two time points for the knee-specific PROMs (R2 range, 0.64–0.93) [22]. These findings were similar to our results in a cohort of elective ACDF patients. Cross et al. prospectively enrolled 153 patients undergoing arthroscopic surgery by orthopedic surgeons to fill out PROMIS forms on the day of surgery which were compared to the PROMIS data routinely collected in clinic [23]. They found no significant difference in PROMIS scores for physical function and upper extremity between preoperative and perioperative setting while pain interference (*p* = 0.042) and depression (*p* = 0.004) were significantly higher on the day of surgery [23]. However, this study is limited by the heterogenous data, which included knee, shoulder, hip, and elbow arthroscopy. Furthermore, the time between the preoperative and perioperative time points varied greatly from less than 14 days to more than 30 days for some patients.

These findings have practical implications for both clinical operations and research design. From a workflow perspective, institutions with limited perioperative staffing or high surgical volume can confidently obtain baseline PROMs in the clinic without sacrificing data accuracy. This flexibility may reduce day-of-surgery bottlenecks and improve patient flow, particularly in centers where anesthesia and nursing teams face competing demands. Furthermore, for centers with higher clinic patient volumes, the ability to administer these surveys in a perioperative setting may improve outpatient clinic workflow, without compromising response accuracy. Additionally, for multicenter studies, standardized PROM collection protocols that allow either setting may facilitate broader participation and improve external validity. Physicians face numerous challenges administering these surveys, and these findings demonstrate that physicians can administer these surveys whenever is convenient for the workflow of their institution [24].

Beyond ACDF, the methodology of this study can inform PROM collection strategies in other subspecialties such as total joint arthroplasty, shoulder arthroscopy, and lumbar fusion. In these fields, as in spine surgery, accurate baseline PROMs are essential for evaluating the effectiveness of surgical interventions, supporting reimbursement models, and guiding shared decision-making [6,25]. Future research should investigate whether these findings hold true for more acute presentations or conditions with rapidly changing symptom profiles, where timing might play a more significant role.

The mental state of patients undergoing spine surgery has been hypothesized to impact their PROMs. While this effect has been difficult to elucidate given the variability in emotional and mental states, spine surgery is a significant operation and can be a stressful and anxious period for many patients. The subset of patients with diagnosed anxiety disorders may have greater susceptibility to the stress of having surgery. A study by Nixon et al. on 205 patients who underwent foot and ankle surgery found that patients with anxiety had greater preoperative pain and lower physical function on the PROMIS scores than patients without anxiety [26]. In a similar study, Goyal et al. studied 391 patients who underwent 1- to 3-level lumbar fusion and found that patients with anxiety and/or depression had greater preoperative Oswestry Disability Index (ODI) scores and lower Short Form-12 (SF-12) scores than patients without either anxiety or depression [27]. Specifically in cervical spine patients with radiculopathy being treated surgically, Skeppholm et al. found in 136 patients with two-year follow-up that those with high preoperative levels of anxiety or depression had poorer baseline data and worse outcomes at follow-up [11]. Attempts to explain these findings include the possibility that patients with anxiety present with more severe symptomatic diseases. However, it is also possible that anxious patients may be uprating their pain and disability. We hypothesized that anxious patients uprate their health status on the day of surgery given the stress of imminent surgery. In our study, patients with anxiety did not have significantly different PROMs between preoperative clinic and day of surgery. This may indicate that the effect of both situational stresses on the day of surgery and pre-existing anxiety on baseline PROMs in this specific patient population is minimal.

This study has several limitations. The primary limitation is the short mean interval between clinic and day-of-surgery PROM collection (mean 7.9 days), which may limit generalizability where preoperative visits occur weeks earlier. The study was not designed or powered to test equivalence at 2 weeks, 1 month, 3 months, or 6 months before surgery, or to evaluate whether anxiety moderates timing effects. These constraints should be considered when applying the findings to other workflows. We did not record whether PROMs were completed at home versus in clinic, nor did we capture the specific administration mode. Because mode can affect data quality and scores, generalizability to remote or mixed-mode workflows is uncertain and warrants dedicated evaluation [28]. We acknowledge the potential for selection bias, which may limit the generalizability of our findings. To minimize this risk, all consecutive patients meeting the eligibility criteria during the study period were approached for participation using a standardized enrollment protocol across all surgeons in our division, and the same enrollment protocol was used across all attending surgeons in our division. Eligibility assessment was based on predefined inclusion and exclusion criteria applied prospectively, and PROM collection followed a standardized workflow in both clinic and perioperative settings. Research staff were trained to offer participation to all eligible patients without deviation, reducing the likelihood of preferential enrollment. However, we did not prospectively maintain a formal screening log documenting the total number of patients approached or the reasons for non-participation. As a result, we cannot retrospectively determine how many patients declined consent or were excluded prior to enrollment. While this uniform recruitment strategy likely reduced bias, the absence of detailed screening data remains a limitation, as it prevents a more precise quantification of the potential impact of selection bias. Moreover, eligibility required English-language literacy, and we did not prospectively track the number excluded for language; this criterion may introduce selection bias and limit generalizability to populations with limited English proficiency. Although non-English versions of several instruments are validated, we did not deploy them. This may introduce selection bias and limit generalizability to patients with limited English proficiency. Barriers included the absence of a multi-language data-capture build, licensing or permission requirements for translated instruments, interpreter staffing constraints on the day of surgery, and insufficient sample size to evaluate cross-language measurement invariance. Although PROMIS, NDI, and mJOA instruments have validated translations in multiple languages, variability in cultural interpretation, health literacy, and the clinical context in which surveys are administered may still influence responses. Including validated non-English instruments with interpreter support in future work can help confirm that, within our specific patient population and workflow observed outcomes are attributable to patient condition rather than subtle linguistic or contextual differences. The completion of demographic information, PROMIS, NDI, mJOA, and VAS took approximately 15–20 min on average, which may induce a degree of survey burden and survey fatigue for some patients. However, the time to complete the PROMs on the day of surgery varied greatly and largely depended on when the patient arrived in the perioperative holding area and the surgical workflow of the nursing and anesthesia staff. Secondly, one of the strengths of this study is that patients served as their own controls from the study design. However, the patients filled out the same set of PROMs for the second time on the day of surgery, which may lead to recall bias. Moreover, use of Bonferroni, while appropriate for familywise control, increases the risk of type II error; small differences may go undetected. Finally, this study may not be generalizable to other elective orthopedic procedures given a variety of other situational factors. Nonetheless, this is a prospective observational study that suggests baseline PROMs can be obtained in either setting, helping guide clinical research using PROMs in spine and orthopedic surgery.

## 5. Conclusions

There was no statistical or meaningful difference between the baseline PROMs obtained from patients receiving ACDF based on the clinical setting. Within about one week before ACDF, clinic and day-of-surgery PROMs appear interchangeably. The finding suggests that surgical centers should determine the ideal workflow to collect PROMs data based on their available resources, as the results demonstrate that the clinical setting has no meaningful impact on the data collected. Future work should test longer preoperative intervals (2 weeks, 1 month, 3 months, 6 months), assess whether administration mode and perioperative anxiety influence baseline reporting, and include validated non-English instruments with interpreter support to confirm comparability across languages.

## Figures and Tables

**Table 1:** Demographic data for all enrolled patients.

Variable	*N* (% or 95% Confidence Interval)
Number of Patients	48
Age	52.4 ± 10.5 (95% CI, 49.4–55.4)
Sex	
Female	28 (58.3%)
Male	20 (41.7%)
Race	
White	36 (75.0%)
Black	11 (22.9%)
Other	1 (2.1%)
Smoking	
Active smoker	8 (16.7%)
Quit smoking	14 (29.2%)
Never Smoked	26 (54.2%)
Body Mass Index (BMI)	31.4 ± 8.1 (95% CI, 29.1–33.8)
Charlson Comorbidity Index (CCI)	1.3 ± 1.4 (95% CI, 0.9–1.7)
Total Number of Surgeries	3.9 ± 4.2(95% CI, 2.7–5.1)
Number of Days Between Baseline Visits (Preoperative Clinic and Day of Surgery)	7.9 ± 5.1 (95% CI, 6.4–9.3)

**Table 2:** Survey results for all enrolled patients.

Variable	Preop Clinic Mean	Preop Clinic Standard Error	Day of Surgery Mean	Day of Surgery Standard Error	*p*-Value *
PROMIS Physical Function	39.2	2.34	39.8	2.14	0.398
PROMIS Pain Interference	64.5	1.98	64.2	1.87	0.682
PROMIS Fatigue	60.5	2.48	58.5	2.42	0.019
PROMIS Social Sat	40.1	2.44	39.2	2.38	0.331
PROMIS Anxiety	60.0	2.73	58.6	2.76	0.047
PROMIS Depression	53.7	2.78	51.7	2.75	0.042
NDI	22.3	1.51	20.8	1.54	0.072
mJOA	14.8	0.43	15.0	0.35	0.566
VAS Neck	6.0	0.41	5.4	0.40	0.054
VAS Body	5.2	0.51	4.8	0.48	0.335

* Following Bonferroni correction for multiple testing, *p* < 0.005 denotes statistically significant.

**Table 3:** Demographic data comparing patients with and without anxiety.

Variable	Patients with Anxiety *	Patients Without Anxiety	*p*-Value
Number of Patients	32	16	
Age	51.4 ± 10.3 (95% CI, 47.7–55.1)	54.4 ± 10.8 (95% CI, 48.7–60.2)	0.255
Sex			0.038
Female	22 (68.8%)	6 (37.5%)	
Male	10 (31.3%)	10 (62.5%)	
Race			0.067
White	22 (68.8%)	14 (87.5%)	
Black	10 (31.3%)	1 (6.3%)	
Other	0	1 (6.3%)	
Smoking			0.341
Active Smoker	7 (21.9%)	1 (6.3%)	
Former Smoker	8 (25.0%)	6 (37.5%)	
Never Smoked	17 (53.1%)	9 (56.3%)	
Body Mass Index (BMI)	32.0 ± 9.0 (95% CI, 28.7–35.2)	30.3 ± 5.9 (95% CI, 27.2–33.4)	0.768
Charlson Comorbidity Index (CCI)	1.0 ± 1.1 (95% CI, 0.6–1.4)	1.9 ± 1.7 (95% CI, 1.1–2.8)	0.063
Total Number of Surgeries	4.2 ± 4.8 (95% CI, 2.5–5.9)	3.3 ± 2.7 (95% CI, 1.8–4.7)	0.599
Number of Days Between Baseline Visits (Preoperative Clinic and Day of Surgery)	7.9 ± 5.9 (95% CI, 5.8–10.0)	7.8 ± 3.4 (95% CI, 6.0–9.5)	0.674

* Anxiety was defined as PROMIS Anxiety ≥ 59.4 from either setting.

**Table 4:** Survey results for patients with anxiety.

Variable	Preop Clinic Mean	Preop Clinic Standard Error	Day of Surgery Mean	Day of Surgery Standard Error	*p*-Value *
PROMIS Physical Function	36.5	2.32	37.3	2.14	0.348
PROMIS Pain Interference	66.6	2.08	66.6	1.91	0.949
PROMIS Fatigue	63.8	2.51	62.3	2.44	0.097
PROMIS Social Sat	37.9	2.51	37.3	2.46	0.628
PROMIS Anxiety	65.1	2.65	63.2	2.71	0.044
PROMIS Depression	57.2	2.57	55.2	2.61	0.034
NDI	26.0	1.74	24.1	1.89	0.079
mJOA	14.2	0.54	14.6	0.45	0.328
VAS Neck	6.6	0.47	6.2	0.46	0.263
VAS Body	6.2	0.62	5.7	0.59	0.301

* Following Bonferroni correction for multiple testing, *p* < 0.005 denotes statistically significant.

## Data Availability

Data is available upon request from authors.

## Abbreviations

The following abbreviations are used in this manuscript:

PROMs	Patient Reported Outcome Measures
ACDF	Anterior Cervical Diskectomy and Fusion
mJOA	Modified Japanese Orthopaedic Association
PROMIS	Patient-Reported Outcomes Measurement Information System
NDI	Neck Disability Index
VAS	Visual Analog Scale
ODI	Oswestry Disability Index
